# Comparison of two statistical indicators in communicating epidemiological results to the population: a randomized study in a high environmental risk area of Italy

**DOI:** 10.1186/s12889-019-7003-y

**Published:** 2019-06-11

**Authors:** Michela Baccini, Laura Ghirardi, Domenica Farinella, Annibale Biggeri

**Affiliations:** 1 Department of Statistics, Computer Science, Applications, University of Florence, Viale Morgagni 59, 50134 Florence, Italy; 2 Department of Medical Epidemiology and Biostatistics, Karolinska Institutet, PO Box 281, SE-171 77 Stockholm, Sweden; 3 Department of Political Sciences and Law, University of Messina, Via T. Cannizzaro 278, 98122 Messina, Italy; 4 Biostatistics Unit, ISPO - Cancer Prevention and Research Institute, Via Cosimo Il Vecchio 2, 50139 Florence, Italy

**Keywords:** Randomized trial, Environmental health, Health impact assessment, Risk communication, Statistical indicators, Time needed to harm, Propensity score

## Abstract

**Background:**

When communicating risks to the general population, the format of the epidemiological results may affect individual reactions. In environmental epidemiology, no study has compared the use of different statistical formats in communicating results to the population. The aim of this paper is to investigate whether the degree of concern expressed by residents of a high environmental risk site, regarding epidemiological results on cancer mortality in the area where they live, is influenced by the statistical indicator used in communication.

**Methods:**

A sample of residents in the high environmental risk area of Livorno (Italy) was randomized to respond to different questionnaires, in which the same epidemiological results were expressed by two alternative risk indexes: percent excess risk and time needed to harm, defined as the number of days that one has to wait for, on average, to observe 1 death in excess in respect to the baseline. Participants were asked to express their concern on a quantitative scale or to rank different diseases according to their impressions. The statistical analysis was performed using an Inverse Probability of Treatment Weighting approach based on propensity score, in order to account for sample stratification and adjust for unbalance between groups occurring despite randomization.

**Results:**

The probability of high concern levels was larger under time needed to harm than under percent excess, with a difference between proportions of 6.7% (95% Confidence Interval, 0.6,12.8%). Mortality from sexual glands cancer was ranked as more worrisome and mortality from thyroid gland cancer as less worrisome under time needed to harm than under percent excess. No rank change was found for lung cancer. Larger differences between the two indicators arose in subjects with higher education or better numerical skills.

**Conclusions:**

Communicating epidemiological results to the population is not a neutral task. The degree of concern and judgments when comparing results on different diseases may depend on the risk indicators used. Translating scientific results into lay language should not exempt from careful evaluation of the impact of this translation on lay people.

**Electronic supplementary material:**

The online version of this article (10.1186/s12889-019-7003-y) contains supplementary material, which is available to authorized users.

## Background

Risk communication is an important but difficult task, which poses numerous challenges for scientists, health practitioners and policy makers. Research has investigated how risk and benefit judgement in different fields may differ between experts and lay people [1, 2], however this discrepancy does not necessarily imply lay people’s higher tendency to misunderstanding or misperceptions [3]. Indeed, it may reflect different perspectives and judgements as a result of several factors influencing the risk evaluation process, including personal attitudes, general knowledge on the subject, media portrayals of hazards and risks, public perceptions, social roles and networks [3–5].

The formatting of the information seems to be an important factor in risk communication. Several studies have investigated how the use of alternative numerical and/or graphical formats to express risks and benefits related to a medical diagnosis or a treatment may influence patient’s decisions, confidence on such decision or level of concern, and two recent systematic reviews have concluded that different formats seem to have an impact on perceived magnitude of the risks [6, 7]. Sensitivity to result format seems to be an important aspect to consider also in the context of public health issues at the population level. This becomes even more important in the context of “community based participatory research” paradigms that call for a collaborative process in which the communities are involved to “co-create knowledge” [8].

In environmental epidemiology, no previous study has compared the use of different statistical formats in communicating results to the general population, despite, especially in environmental emergency contexts, communication plays a crucial role. Hence, with the present paper we aim at investigating whether the degree of concern expressed by residents of a high-risk area about epidemiological results on cancer mortality in the same area is influenced by the statistical indicator used to communicate such results [9]. In particular, we report the results of a randomized study conducted on a sample of residents in the city of Livorno (159,431 inhabitants - western coast of Tuscany), which, together with the neighboring municipality of Collesalvetti (16,791 inhabitants), is classified as a high-risk environmental site, according to the Seveso Directive [10–12], due to the presence of a large commercial harbor and several petrochemical plants producing dangerous pollutants [13]. According to what frequently done in similar studies performed in clinical context [7], the randomized study compared a relative risk indicator with an absolute risk indicator, when used to communicate results about the health profile of the population in the area of interest (see Methods).

## Methods

### Participants

A sample of 579 residents aged between 18 and 80, stratified by gender, age (18–30, 30–40, 40–50, 50–60, 60–70, over 70) and urban district (5 urban districts), was randomly extracted from the municipality records of Livorno. The sample size was established accounting for feasibility and statistical considerations (see Additional file 1). Then, subjects within each stratum were randomly assigned to one of 3 trained interviewers. Within each stratum defined on age, gender, district and interviewer, subjects were randomized to one of the different indicators under comparison. Interviews were collected from October 2012 to March 2013. Of the initial sample of 579 inhabitants, 340 responded to the questionnaire (59%). The response rate was similar for males and females, and lower in young people (18–25) and over 75 than in the other age groups. The urban district 5, in the South of the city, was characterized by the lowest response rate.

The study was performed within a project funded by the Istituto Toscano Tumori and approved by the local ethics committee on September 2010.

### Indicators under comparison

Depending on the randomization arm, the burden of mortality attributable to" living in Livorno-Collesalvetti" was expressed through one of the following two indicators:

1) the percent excess of risk (% excess) of death in Livorno-Collesalvetti in respect to Tuscany:

$$ \%\kern0.5em excess=100\ast \left(O-E\right)/E=100\ast \left( SMR-1\right) $$

where O was the observed number of deaths from a specific cause in the area during the period of interest, E was the corresponding expected number of deaths, calculated according to the regional rates by age, gender and deprivation level, and SMR was the standardized mortality ratio, calculated as the ratio O/E;

2) the time needed to harm (TNH), i.e. the number of days one has to wait for, on average, to observe 1 death in excess in Livorno, taking Tuscany as the reference:

$$ TNH=N/\left(O\ast \left(1-1/ SMR\right)\right) $$

where N is the total follow up duration, in days.

While the percent excess represents a relative measure of excess mortality, the proposed TNH is an absolute measure of excess mortality. This indicator is similar to the Number Needed to Harm (NNH), which is conventionally used to express risks associated with a treatment and refers to the number of individuals receiving the treatment needed to have an additional adverse event. As for NNH, the smaller the TNH, the higher is the impact. Quantifying impacts in terms of time needed to observe an event is quite usual in communicating epidemiological results and examples can be found also in clinical context ([14], https://www.theguardian.com/society/2016/jan/20/older-person-dying-winter-fuel-poverty, [15])

With the aim to provide sufficient information for deriving the absolute excess of mortality in both arms, we always accompanied the percent excess with the total number of deaths observed in the study area.

### Questionnaires

Questionnaires development started from a preliminary draft, which was assessed on a small sample of residents through the cognitive interviewing technique [9]. For simplicity, we will refer to the version of the questionnaire where the burden of mortality was expressed in terms of % excess as % excess-questionnaire, and to the version where the burden was expressed in terms of TNH as TNH-questionnaire.

Under both experimental conditions, participants had to rate their degree of concern about mortality from cancer in Livorno in respect to the regional average on a scale from 1 to 10 (item R3). Then, results regarding mortality from three different types of cancer (sexual glands cancer, thyroid cancer, lung cancer) among women were presented, and participants were asked which one was the most and the least alarming option (item R4). The formulation of questions R3 and R4 differed under the two experimental conditions (Table 1). For example, in Livorno during the reference period there were 620 deaths from cancer, corresponding to a SMR equal to 104.5%. This result was expressed in terms of a 4.5% excess in mortality from cancer in the % excess-questionnaire (coupled with the total number of deaths from cancer), and in terms of one extra death from cancer every 13 days in the TNH-questionnaire, in both cases taking the mortality level in the region as reference. It is worth noticing that the enrolled subjects were expected not to be more familiar with one of the two indicators than with the other, because no information campaign was been conducted before the randomized experiment.

**Table 1:** Questions R3 and R4, aimed to compare the degree of concern of respondents when the same results were expressed in terms of % excess and Time Needed to Harm (TNH). English translation from the Italian

Question	Indicator^a^	
R3	% excess	From 2001 to 2006, death rates from cancer in Livorno-Collesalvetti were 4.5% higher than in Tuscany as a whole, with 620 overall deaths every year.
		Please state your concern about this result on a scale ranging from 0 (no concern) to 10 (extremely concerned).
	TNH	From 2001 to 2006, we observed 1 more death from cancer every 13 day in Livorno-Collesalvetti relative to Tuscany as a whole.
		Please state your concern about this result on a scale ranging from 0 (no concern) to 10 (extremely concerned).
R4	% excess	From 2001 to 2006, we observed the following results in Livorno-Collesalvetti*: a-The risk of death from sexual glands is 25% higher than in Tuscany, with an overall number of 13 deaths every year.
		b- The risk of death from thyroid cancer is 60% higher than in Tuscany, with an overall number of 2 deaths every year.
		c- The risk of death from lung cancer is 4% higher than in Tuscany, with an overall number of 27 deaths every year.
		Please, tick which result is the most concerning and which one is the least concerning to you.
		(*) results refer to women.
	TNH	From 2001 to 2006 we observed the following results in Livorno-Collesalvetti*:
		a-one more death every 4 months and a half relative to Tuscany from sexual glands cancer.
		b-one more death every 14 months and a half relative to Tuscany from thyroid cancer.
		c- one more death every 12 months, relative to Tuscany from lung cancer.
		Please, tick which result is the most concerning and which one is the least concerning to you.
		(*) results refer to women.

Notes: ^a^The average numbers of excess deaths reported in the questions are rounded to the closest integer

The questionnaires contained also items assessing baseline attitude towards risk (question R1), baseline risk perception (question R2), numerical skills and socio-demographic data. While risk attitude and perception were measured at the beginning of the interview, before the questions involving the two indicators, numerical skill and socio-demographic data were collected at the end of the interview. Baseline risk attitude and perception were measured by items concerning the health/safety domain drawn from the Original 40-Item Domain-Specific Risk-Taking (DOSPERT) Scale 2002 (see Additional file 2) [16, 17]. Numerical skills were measured through three open questions concerning probability, derived from Schwartz et al. (1997) [18] (Additional file 3).

### Outcomes

In this paper, we analysed the following outcome variables:

- Degree of concern about mortality from cancer measured on a scale from 1 to 10 from question R3.
- Proportion of subjects who expressed a degree of concern larger than 5 in question R3.
- Rank associated to the concern about mortality from each of the three causes compared in question R4: from 1 (the most worrisome option) to 3 (the least worrisome option).

We a priori selected 5 as the cut-off for the degree of concern about mortality from cancer because it was the intermediate value of the scale. However, a sensitivity analysis was conducted changing the threshold used for the definition of the binary variable.

### Statistical analysis

We analysed data using an Inverse Probability of Treatment Weighting (IPTW) approach based on propensity score (PS). This method attempts to weight individuals according to PS in order to create a ‘pseudo-population’ where baseline covariates are balanced between groups [19]. IPTW not only removes possible sources of residual confounding, but also allows us to account for data correlation introduced by stratified randomization. In addition, it may bring to efficiency gain as compared with the regression-based approach, if uncertainty around PS estimates is taken into account [20]. We implemented the IPTW approach using the command *teffects ipw* in Stata 13.1 [21].

In our study, PS was defined as the conditional probability of being assigned to TNH-questionnaire, given the subject’s baseline characteristics. We estimated PS by specifying a logistic model for the questionnaire assignment given the following explanatory variables: age (18–34, 35–64, 65 and over), gender, urban district, interviewer, educational attainment (intermediate school diploma or lower, high school diploma, university degree), numerical skills (at least one right answer over three, no right answer; see Additional file 3), smoking status (current smoker, former smoker, no smoker), employment status (employed, retired, not employed), general risk attitude and risk perception, respectively measured as mean values of the 8 items of questions R1 and R2 (see Additional file 2).

The analyses were conducted excluding the subjects with missing values on the outcome: 9 participants for question R3 (2.6%) and 8 participants for question R4 (2.3%). In order to deal with missing values in the baseline characteristics, for each incomplete explanatory variable, we included in the PS an indicator of missing entry [22].

Relaying on the fact that PS is a balancing score (i.e., conditionally on PS, the distribution of the measured covariates is similar between groups), we evaluated the appropriateness of our PS model by comparing, for each covariate, the between groups standardized mean differences calculated before and after adjustment [23]. We also checked for the overlap of the PS distributions under % excess and TNH, and we removed from the analysis the units not included in the common support.

The relative effect of the two risk indicators was measured in terms of mean differences when degree of concern or ranks were considered, and in terms of proportion differences when considering R3 (“degree of concern for cancer mortality”) as a binary variable (high vs low concern). Stratified analyses were performed by educational achievement (intermediate school diploma; high school diploma or higher) and numerical skills (at least one right answer over three, no right answer; see Additional file 3).

In reporting the results for question R4 (“which result is the most concerning and which one is the least concerning to you”), we draw descriptive cumulative rankograms and we calculated the crude and the adjusted Surfaces Under the Cumulative Rank curve (SUCRA) [24].

It is worth noticing that, despite of the fact that the distribution of the degree of concern for cancer mortality was skewed (see the next section), we focused on the mean difference between groups, in order to enhance the interpretation of the result. However, in a sensitivity analysis based on quantile regression, we performed also a comparison between groups at different quantile of the score (0.1, 0.2, 0.3, 0.4, 0.5, 0.6, 0.7), adjusting for the same variables included in the propensity score model.

## Results

The main characteristics of the 340 respondents, including socio-demographic variables and other covariates included in the PS model, are reported in Additional file 4: Table A1.

The average degree of concern about overall cancer mortality (question R3) was high, equal to 8.30 (on a 1–10 scale), with standard deviation equal to 1.92. Around 91% of sample expressed a degree of concern higher than 5 (see Additional file 5: Figure A1). Notably, 8 and 10 modalities of question R3 received an unexpected amount of preference in respect to 9. Regarding question R4 (“which result is the most concerning and which one is the least concerning to you”), most of respondents declared to be particularly concerned about lung cancer mortality (62.9%), while thyroid cancer was selected as the item causing the lowest concern by 48.8% of the interviewees.

Weighting using propensity score improved covariates balance, in particular for interviewer indicator and risk attitude (see Additional file 6: Figure A2). Few units were excluded from the analysis in order to guarantee overlapping between the PS distributions under % excess and under TNH.

Tables 2 and 3 report the estimated effect of TNH-questionnaire versus % excess-questionnaire on each outcome variable (Average Causal Effect, ACE) and the weighted mean of the outcome, had all individuals been assigned to the % excess-questionnaire, considered here the control condition.

**Table 2:** Degree of concern for cancer mortality: causal effect of expressing the result in terms of TNH versus % excess (question R3)

R3 as a continuous variable: Degree of concern from 1 to 10						
		ACE Mean difference	95% CI	*p*-value	PO % excess	95% CI
	All responders	0.31	(−0.09; 0.70)	0.128	8.15	(7.85 8.44)
R3 as a binary variable: Degree of concern larger than 5						
		ACE Difference of proportions	95% CI	*p*-value	PO % excess	95% CI
	All responders	0.060	(−0.000; 0.121)	0.051	0.880	(0.831; 0.928)
Mathematical skills	Low mathematical skills^a^	−0.067	(−0.221; 0.088)	0.399	0.921	(0.880; 1.002)
	High mathematical skills^a^	0.075	(0.006; 0.144)	0.033	0.871	(0.813; 0.928)
Education level	Intermediate school diploma or lower	0.019	(−0.061; 0.099)	0.644	0.924	(0.866; 0.982)
	High school diploma or higher	0.091	(0.014; 0.168)	0.020	0.862	(0.795; 0.928)

Abbreviations: *ACE* Average causal effect, *95% CI* 95% Confidence Interval, *PO % excess* Potential outcome under the % excess questionnaire

Notes: ^a^Low mathematical skills: No right answer at questions evaluating numerical skills; High mathematical skills: At least one right answer at questions evaluating numerical skills

**Table 3:** Ranking sexual gland cancer, thyroid cancer and lung cancer mortalities according to the degree of concern (from 1: high concern, to 3: low concern): causal effect of expressing the results in terms of TNH versus % excess (question R4)

	ACE Mean difference	95% CI	*p*-value	PO % excess	95% CI	Adjusted SUCRA % excess	Adjusted SUCRA TNH
Rank assigned to sexual glands cancer mortality	−0.13	(−0.28; 0.02)	0.093	2.26	(2.16; 2.35)	0.370	0.435
Rank assigned to thyroid cancer mortality	0.19	(0.03; 0.35)	0.021	2.24	(2.11; 2.36)	0.380	0.285
Rank assigned to lung cancer mortality	−0.06	(−0.21; 0.09)	0.432	1.51	(1.39; 1.62)	0.745	0.775

Abbreviations *ACE* Average causal effect, *95% CI* 95% Confidence Interval, *PO % excess* Potential outcome under the % excess questionnaire, *Adjusted SUCRA* Surface under the cumulative ranking curve from the Inverse Probability of Treatment Weighting  model

As reported in Table 2, when epidemiological results were expressed in terms of TNH, the average concern about cancer mortality due to “living in Livorno” increased by 0.31 units (*p* = 0.128), on a 1 to 10 scale, being the potential outcome mean under % excess equal to 8.15. Correspondently, the percentage of subjects declaring a concern larger than 5 increased by 6% (*p* = 0.051) under TNH, being the percentage under % excess equal to 88%.

The sensitivity analysis based on quantile regression provided results consistent with the estimated mean difference of 0.31 units arising from the main analysis (see Additional file 7: Figure A3). Analogously, using different cut-offs for the degree of concern (from 2 to 4 and from 6 to 9), the estimated probability of scores above the threshold still remained larger under TNH than under % excess, although the uncertainty of the results increased with higher cut-offs, reflecting the fact that, by increasing the cut-off, the probability of scores above the threshold progressively approached 50% (see Additional file 8: Figure A4).

**Fig. 1:**
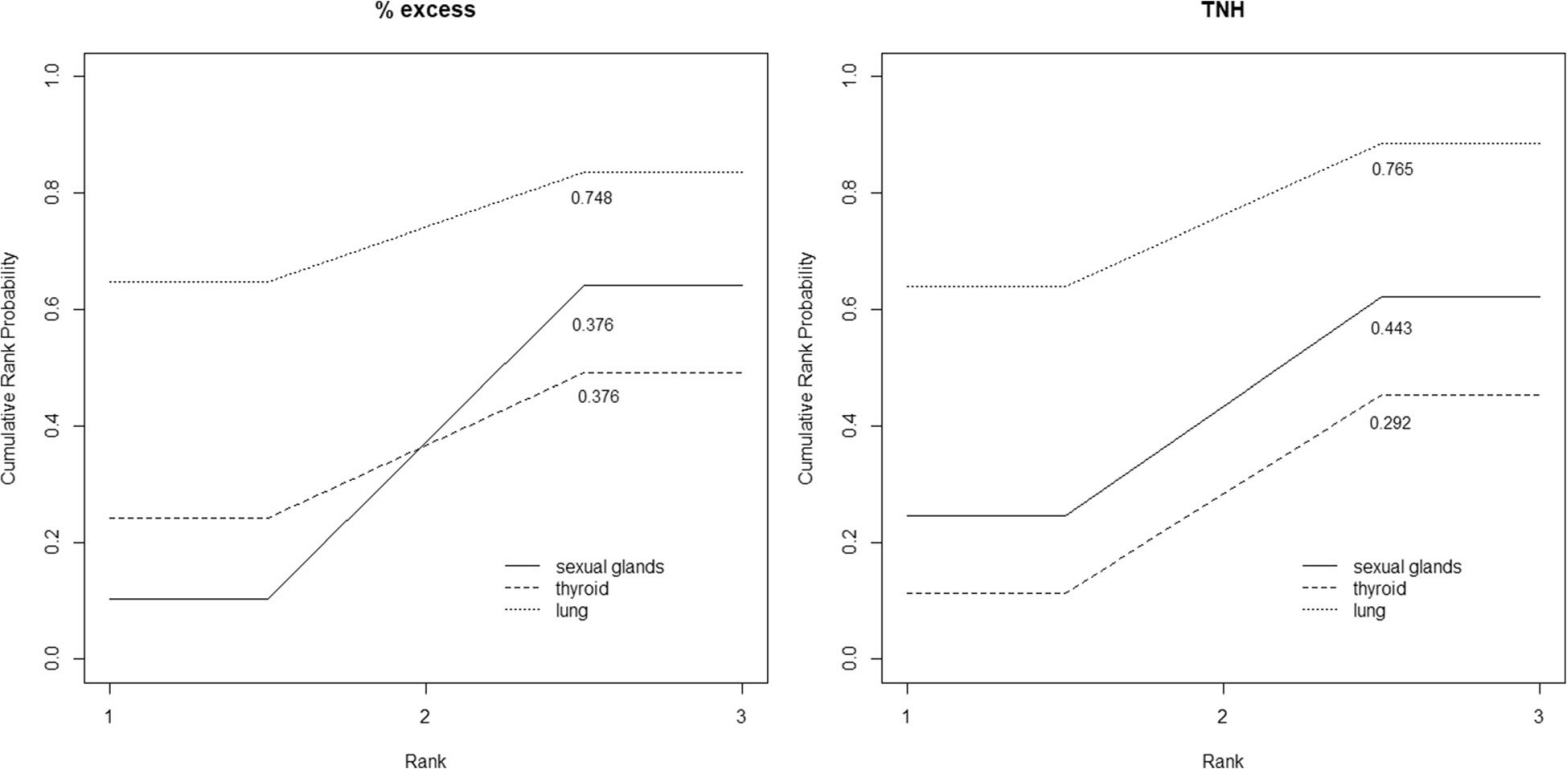
Cumulative rankograms under % excess and TNH. Cumulative rankograms for sexual glands cancer, thyroid cancer and lung cancer mortalities, under % excess and TNH. The proportion of surface under each curve (SUCRA) is also reported

Figure 1 reports the cumulative rankograms for the three causes of deaths compared in question R4 (“which result is the most concerning and which one is the least concerning to you”), under % excess and TNH. The cumulative rankogram for a specific cause of death represents the probabilities that that cause is classified among the *k* most worrisome ones, where *k* ranges from one to three. For each cumulative rankogram, we calculated also the surface under it, so-called SUCRA, which can be used to define a hierarchy among the three causes of deaths, with larger SUCRA values indicating higher degree of concern [24]. While judgement about lung cancer mortality, classified as the most worrisome item, was similar under the two indicators (SUCRA = 0.748 and 0.765 under % excess and TNH, respectively), a certain difference was observed for thyroid cancer and sexual glands cancer. The adjusted analyses confirmed these descriptive results (Table 3). No significant change in rank was observed for lung cancer mortality, while, compared with % excess, TNH caused sexual glands cancer mortality to be rated as more severe (difference between average ranks = − 0.13; *p* = 0.093) and thyroid gland cancer mortality to be rated as less worrisome (difference between average ranks = 0.19; *p* = 0.021). The adjusted SUCRA values, obtained as a simple transformation of the average ranks arising from IPTW regressions, were very similar to the unadjusted ones (Table 3).

Among people with high school diploma or higher, the probability of expressing degree of concern greater than 5 increased by 9.1% (*p* = 0.020) under TNH, taking % excess as reference (Table 2). Among people with intermediate school diploma there was no evidence of a difference between the two indicators. In the subgroup with higher mathematical skills, the probability of declaring a degree of concern greater than 5 increased by 7.5% (*p* = 0.033) under TNH, taking % excess as reference. Among people with low mathematical skills, the same ACE estimate was negative and affected by large variability (Table 2).

**Fig. 2:**
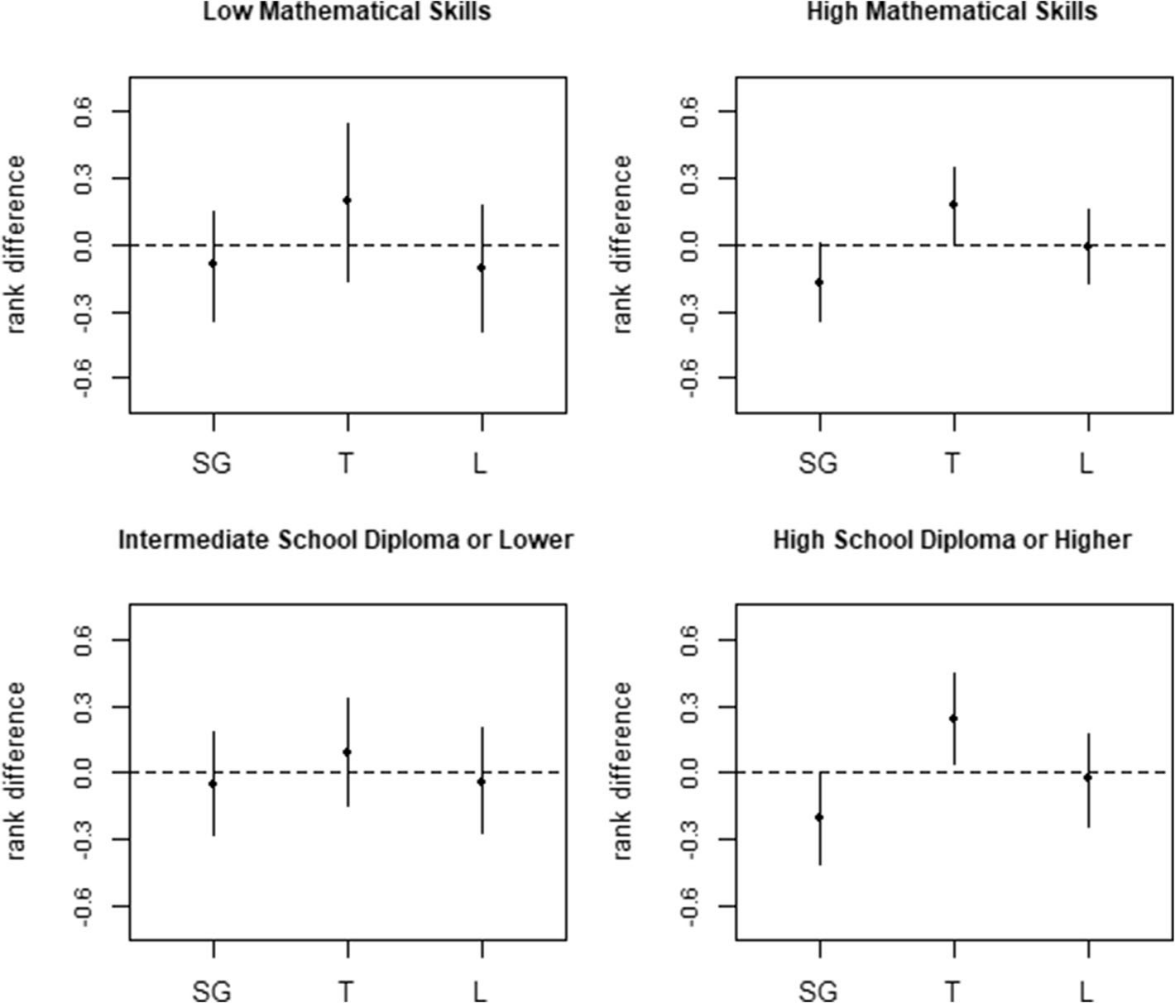
Mean rank differences between % excess and TNH, by mathematical skills and education level. Differences in ranking sexual gland cancer (SG), thyroid cancer (T) and lung cancer (L) mortalities according to the degree of concern (from 1: high concern, to 3: low concern), when comparing TNH and % excess, are expressed in terms of mean rank differences, with 95% Confidence Intervals. The analyses, based on the IPTW approach, were performed by mathematical skills (Low mathematical skills: No right answer at questions evaluating numerical skills; High mathematical skills: At least one right answer at question evaluating numerical skills) and education level (Intermediate school diploma or lower; High school diploma or higher)

As shown in Fig. 2, the ranks assigned to the three causes of deaths in question R4 by the respondents with intermediate school diploma or lower mathematical skills did not depend on the statistical indicator used. On the contrary, an effect of the indicator was found among the respondents with higher educational level or higher mathematical skills, limited to the ranks assigned to sexual gland cancer and thyroid cancer (for details see Additional file 9).

## Discussion

In clinical context, several studies compared the use of alternative indicators of relative risk and absolute risk in communication to patients [7, 25]. This is the first study that tries to make something similar in the field of environmental epidemiology, with a randomized experiment in an area at high environmental risk. A sample of citizens was informed about epidemiological results derived from scientific work conducted on their own area. We focused on the individual degree of concern induced by communicating epidemiological results using two alternative risk indicators; investigating the level of understanding of the numerical messages by the respondents was out of the aims of our study.

We found that judgments about local risks for population health were influenced by how these risks were communicated. Specifically, a measure similar to the Number Needed to Harm, which we called Time Needed to Harm, appeared to cause slightly greater concern in citizens than % excess risk, when the overall result concerning mortality from different types of cancer was communicated.

Expressing the impact in terms of TNH led people to rank mortality from sexual glands cancer as slightly more alarming and mortality from thyroid gland cancer as slightly less alarming, as compared with expressing the same results in terms of % excess. On the contrary, no change was evident for the relative judgment about lung cancer, which was ranked as the most worrisome disease under both the experimental conditions. This result may be due to a complex mixture of factors that go beyond the numbers communicated, including role of health information campaigns and people’s knowledge, experience and personal view about severity and curability of the three diseases, and about factors than can cause them.

Educational level and numeracy influenced health risk evaluation, confirming evidence reported elsewhere [26, 27]. In particular, the observed differences between indicators were larger when the subject had higher education or better numerical skills. A possible explanation of this result is that people with higher education level/mathematical skills tend to pay more attention to numbers, as compared with people with lower educational level/mathematical skills. On the contrary, the latter group may be more influenced by other factors, for example personal experiences and views.

The role of individual experience in assigning scores or ranking diseases had emerged as relevant also during the cognitive interviews conducted to build the questionnaires: people tended to consider as more worrisome those diseases that they experienced directly or indirectly during their life, and the responses were sometimes influenced by the personal knowledge on the environmental pollution in the city and its relationship with specific diseases [28]. For this reason, even if investigating the role of individual experience of the interviewees with the diseases mentioned in the questionnaire was out of our aim, we introduced in the final questionnaires a close-ended question to investigate the possible reasons of the response to R4 (“which result is the most concerning and which one is the least concerning to you”) [9]. Only 32% of the 340 respondents declared to have replied on the basis of the numerical data presented, while the remaining 68% declared to have replied on the basis of personal knowledge or experience about the illness (37%) or on the basis of personal knowledge about pollutants released in the study area (31%).

### Study limitations

In this research, the non-response rate was around 41%. This is in line with the rate of non-response in Italian surveys, which ranges between 20 and 50% [29]. In our study, non-response was probably due to the difficulty in contacting potential participants using the municipality registry and in doing interviews at home (change of address, difficulty to find people at work or at school). Another plausible reason was the complexity of the questionnaires and the sensitive research topic (health status of the resident population) [9]. Even the lack of trust in institutions may have induced people to avoid participation, but this aspect was not addressed in this study. As a consequence of the large non-response rate, the respondents might represent a selected subgroup of the original sample, with possible impact on the generalizability of the results in the presence of an interaction effect between type of indicator and factors related to the non-response. However, because of the randomized nature of the experiment, it is likely that this selection did not bring to biased effect estimates.

A second limitation concerns the outcome that we measured. In fact, due to the complex and multidimensional nature of risk perception, we focused only on a specific aspect of this construct, that is the degree of concern of the respondents [30]. Similarly, this study does not to provide an exhaustive comparison among alternative numerical formats, being % excess and TNH only two of the possible indicators to be used for communicating epidemiological results.

From a statistical point of view, the comparison between the two indicators was made complex by the fact that the distribution of the outcome variable measuring the degree of concern of the respondents was strongly asymmetric. We performed several sensitivity analyses, which confirmed the robustness of our result, but for future investigations a revision of the response scale should be considered.

Finally, the literature reports a variety of sociocultural, economic and psychological factors being crucial in modelling judgements and decisions [5, 31]. Detecting these factors is important to facilitate public health communication and promote equal access to information across society. In the present study, we conducted only subgroup analyses according to educational level and numeracy. Future studies aimed at elucidating the role of these factors may benefit from a more extensive assessment of the individual characteristics.

## Conclusions

Our experiment shows that communicating epidemiological results to the population is not a neutral task. In fact, the degree of concern induced by the presentation of results about community health, as well as the ranking of concern when comparing the results on different diseases, may depend on the risk indicator used.

Additionally, we found that the impact of using different numerical formats may vary according to individual characteristics, such as education level or mathematical skill. In particular, people having higher education level/mathematical skills seem to be more influenced by the numerical formats of the message than people with lower education level/mathematical skills, who tend to express the same degree of concern independently from the statistical indicator used in communication. This likely originates from a different ability of the message to reach different individuals: the higher the actual or perceived ability to understand numbers, the more attention is given to the numerical content of the message; the lower the actual or perceived ability to understand numbers, the higher is the role of the a priori knowledge in interpreting the message. This result supports the idea that ignoring such factors, that may hamper or facilitate communication of health risks, can lead to unequal information and, as a consequence, unequal protection/prevention across society [32]. Therefore, communication strategies shared by different actors are needed, which account for the heterogeneity of the population to whom the message is addressed.

## Additional files

Additional file 1: Sample size determination. (PDF 42 kb)

Additional file 2: Questions R1 and R2. Description of questions R1 and R2 on risk attitude and perception. (PDF 15 kb)

Additional file 3: Question on numerical skills. Description of the question on numerical skills. (PDF 33 kb)

Additional file 4: **Table A1.** Description of the sample: socio-demographic characteristics, smoking habit, numerical skills, baseline risk perception and baseline attitude toward risk. (PDF 41 kb)

Additional file 5: **Figure A1.** Histogram of the degree of concern of the respondents from question R3. (PDF 11 kb)

Additional file 6: **Figure A2.** Standardized differences between the two experimental groups for each covariate included in the Propensity Score model, before and after adjustment through Inverse Probability of Treatment Weighting. (PDF 38 kb)

Additional file 7: **Figure A3.** Degree of concern for cancer mortality: estimated differences between TNH and % excess at the quantiles of the outcome distribution (*p* = 0.1, 0.2, 0.3, 0.4, 0.5, 0.6, 0.7), and corresponding 95% confidence intervals (question R3). (PDF 43 kb)

Additional file 8: **Figure A4.** Degree of concern for cancer mortality: estimated differences between the probabilities of a degree of concern larger than the cut-off under TNH and under % excess (cut-off = 2, 3, …, 9), with the corresponding 95% confidence intervals (question R3). (PDF 43 kb)

Additional file 9: **Table A2.** Ranking sexual gland cancer, thyroid cancer and lung cancer mortalities according to the degree of concern (from 1: high concern, to 3: low concern). Causal effect of expressing the results in terms of TNH versus % excess (question R4), by education level and mathematical skills. (PDF 18 kb)

## Data Availability

The datasets analyzed during the current study are available from the corresponding author on reasonable request.

## Abbreviations

ACE: Average Causal Effect
CI: Confidence Interval
IPTW: Inverse Probability of Treatment Weighting
NNH: Number Needed to Harm
PO: Potential Outcome
PS: Propensity Score
SMR: Standardized Mortality Ratio
SUCRA: Surface Under the Cumulative RAnking line
TNH: Time Needed to Harm
