# Supplementary material for: Comparison of two statistical indicators in communicating epidemiological results to the population: a randomized study in a high environmental risk area of Italy
Source: BMC Public Health. 2019 Jun 11;19:733. doi: 10.1186/s12889-019-7003-y (PMC6560769; doi:10.1186/s12889-019-7003-y)
Supplement: Supplementary file 1 — Sample size determination. (PDF 42 kb) [file 12889_2019_7003_MOESM1_ESM.pdf]

Sample size determination

The main aim of the project, in the form approved by the ethics committee, was to investigate the degree of concern in the population through simple questions, with the randomized experiment as secondary outcome. Therefore, the minimum sample size was determined to obtain a 95% confidence interval of width 0.10 on a prevalence estimate of 50%, calculated for a generic binary variable (e.g. prevalence of participants declaring a degree of concern higher than a threshold). According to this choice, the minimum number of subjects required was 390 [1]. Taking into account for a possible non response rate ranging from 30% to 35%, we finally planned to extract a sample of 579 residents. With focus on the randomized experiment and in particular on the between group comparison of the average level of concern measured on a 1-10 scale (question R3), this sample size (290 subjects per arm), would have been sufficient to detect a difference (*d*) of 0.5 when true with a probability of 85% (power), fixing α=0.05 and assuming a standard deviation (*sd*) in the two groups equal to 2. Due to the fact that we had no information about *d* and *sd* from previous published studies, we also evaluated the power for different values of *sd* and *d*, as reported in the figures below.

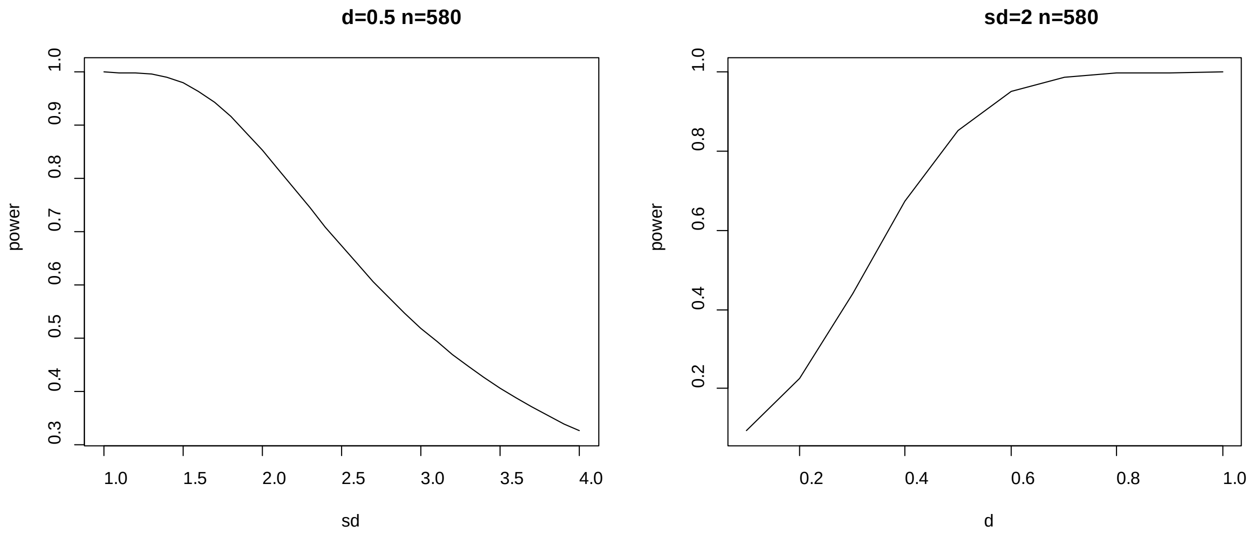

An evaluation of the impact of non response was also done. Fixed *d*=0.5 and *sd*=2, nonresponse rates of 10%, 30% and 40% were expected to lower the power to 0.81, 0.71 and 0.65, respectively.

References

1 Fleiss L, Levin B, Paik MC. Statistical Methods for Rates and Proportions. Third Edition. New York: Wiley; 2003.
