# Supplementary material for: Comparison of two statistical indicators in communicating epidemiological results to the population: a randomized study in a high environmental risk area of Italy
Source: BMC Public Health. 2019 Jun 11;19:733. doi: 10.1186/s12889-019-7003-y (PMC6560769; doi:10.1186/s12889-019-7003-y)
Supplement: Supplementary file 2 — Questions R1 and R2. Description of questions R1 and R2 on risk attitude and perception. (PDF 15 kb) [file 12889_2019_7003_MOESM2_ESM.pdf]

Questions R1 and R2

Questions R1 and R2 on risk attitude and perception were drawn from the Original 40-Item Domain-Specific Risk-Taking (DOSPERT) Scale 2002 [1, 2]. DOSPERT is a psychometric scale that assesses risk attitude and perception in five content domains: financial decisions, health/safety, recreational, ethical, and social decisions. First, respondents rate the likelihood that they would engage in domain-specific risky activities, then, they rate the perception of the magnitude of the expected risks associated to the same activities. In our questionnaire, we included only the 8 items relative to the health/safety domain. Respondents were requested to rate their likelihood/perception on a 7-point scale ranging from 1 to 7.

Question R1

For each of the following statements, please indicate how risky you perceive each situation.

1	2	3	4	5	6	7
Not at all Risky	Slightly Risky	Somewhat Risky	Moderately Risky	Risky	Very Risky	Extremely Risky

Question R2

For each of the following statements, please indicate your likelihood of engaging in each activity or behavior.

1	2	3	4	5	6	7
Extremely Unlikely	Moderately Unlikely	Somewhat Unlikely	Not Sure	Somewhat Likely	Moderately Likely	Extremely Likely

1. Buying an illegal drug for your own use.
2. Consuming five or more servings of alcohol in a single evening.
3. Engaging in unprotected sex.
4. Not wearing a seatbelt when being a passenger in the front seat.
5. Not wearing a helmet when riding a motorcycle.
6. Exposing yourself to the sun without using sunscreen.
7. Walking home alone at night in a somewhat unsafe area of town.
8. Regularly eating high cholesterol foods.

References

1. Weber EU, Blais AR, Betz NE. A domain specific risk attitude scale: Measuring risk perceptions and risk behaviors. J Behav Dec Making. 2002;15:263-90.

2. Blais AR, Weber EU. A domain-specific risk-taking (DOSPERT) scale for adult populations. Judgm Decis Mak. 2006;1:33-47.
