# Supplementary material for: Comparison of two statistical indicators in communicating epidemiological results to the population: a randomized study in a high environmental risk area of Italy
Source: BMC Public Health. 2019 Jun 11;19:733. doi: 10.1186/s12889-019-7003-y (PMC6560769; doi:10.1186/s12889-019-7003-y)
Supplement: Supplementary file 3 — Question on numerical skills. Description of the question on numerical skills. (PDF 33 kb) [file 12889_2019_7003_MOESM3_ESM.pdf]

Question on numerical skills

Question on numerical skills was derived from Schwartz et al. (1997) [1]. It consisted in 3 open questions aimed to assess the numerical skills of respondents.

COMP_1 – Taking a medication, the chance of developing an allergic reaction is 1%. What is your best guess about how many people would develop an allergic reaction if 1,000 people take the medication?

COMP_2 – Taking a medication, the chance of developing an allergic reaction is 1 in 1,000. What percentage of treated patients develop an allergic reaction?

COMP_3 – Imagine that we flip a fair coin 1,000 time. What is your best guess about how many times the coin would come up heads?

References

1. Schwartz LM1, Woloshin S, Black WC, Welch HG. The role of numeracy in understanding the benefit of screening mammography. Ann Intern Med. 1997;127:966-72.
