# Supplementary material for: Comparison of two statistical indicators in communicating epidemiological results to the population: a randomized study in a high environmental risk area of Italy
Source: BMC Public Health. 2019 Jun 11;19:733. doi: 10.1186/s12889-019-7003-y (PMC6560769; doi:10.1186/s12889-019-7003-y)
Supplement: Supplementary file 4 — Table A1. Description of the sample: socio-demographic characteristics, smoking habit, numerical skills, baseline risk perception and baseline attitude toward risk. (PDF 41 kb) [file 12889_2019_7003_MOESM4_ESM.pdf]

Table A1. Description of the sample: socio-demographic characteristics, smoking habit, numerical skills, baseline risk perception and baseline attitude toward risk.

**Variable**		**Frequency**	**Percentage**
**Age**	18-34	73	21.5
	35-64	165	48.5
	>64	101	29.7
	Missing	1	0.3
**Gender**	Female	168	49.4
	Male	170	50.0
	Missing	2	0.6
**Education**	Intermediate school diploma or lower	139	40.9
	High school diploma	140	41.2
	University degree	58	17.0
	Missing	3	0.9
**Employment Status**	Unoccupied	89	26.2
	Occupied	147	43.2
	Retired	100	29.4
	Missing	4	1.2
**Smoking Status**	No Smoker	192	56.4
	Smoker	68	20.0
	Former Smoker	74	21.8
	Missing	6	1.8
**Numerical Skills**	At least one right answer	253	74.4
	No right answer	68	20.0
	Missing	19	5.6
		**Mean**	**Standard deviation**
**Risk Attitude**	Scale: 1 (minimum attitude) to 7 (maximum attitude)	1.99	0.87
	Missing	-	
**Risk Perception**	Scale: 1 (minimum perception) to 7 (maximum perception)	5.92	0.86
	Missing	-	
