# Supplementary figures and images for: Comparison of two statistical indicators in communicating epidemiological results to the population: a randomized study in a high environmental risk area of Italy
Source: BMC Public Health. 2019 Jun 11;19:733. doi: 10.1186/s12889-019-7003-y (PMC6560769; doi:10.1186/s12889-019-7003-y)

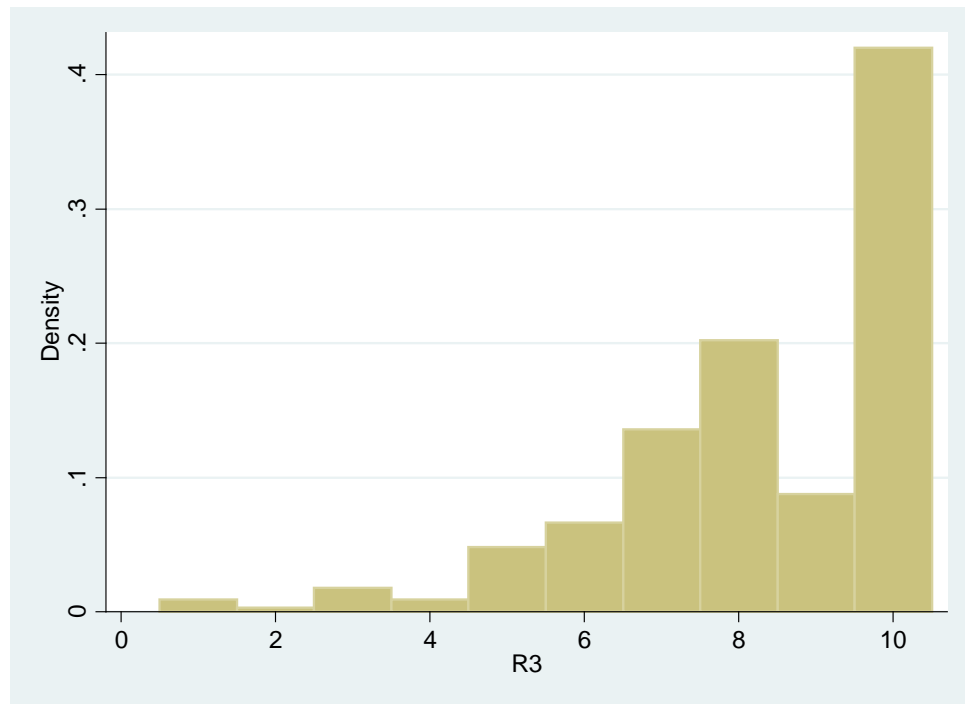

Figure A1. Histogram of the degree of concern of the respondents (question R3).

Supplement: Supplementary file 5 — Figure A1. Histogram of the degree of concern of the respondents from question R3. (PDF 11 kb) [file 12889_2019_7003_MOESM5_ESM.pdf]
