# Supplementary material for: Comparison of two statistical indicators in communicating epidemiological results to the population: a randomized study in a high environmental risk area of Italy
Source: BMC Public Health. 2019 Jun 11;19:733. doi: 10.1186/s12889-019-7003-y (PMC6560769; doi:10.1186/s12889-019-7003-y)
Supplement: Supplementary file 6 — Figure A2. Standardized differences between the two experimental groups for each covariate included in the Propensity Score model, before and after adjustment through Inverse Probability of Treatment Weighting. (PDF 38 kb) [file 12889_2019_7003_MOESM6_ESM.pdf]

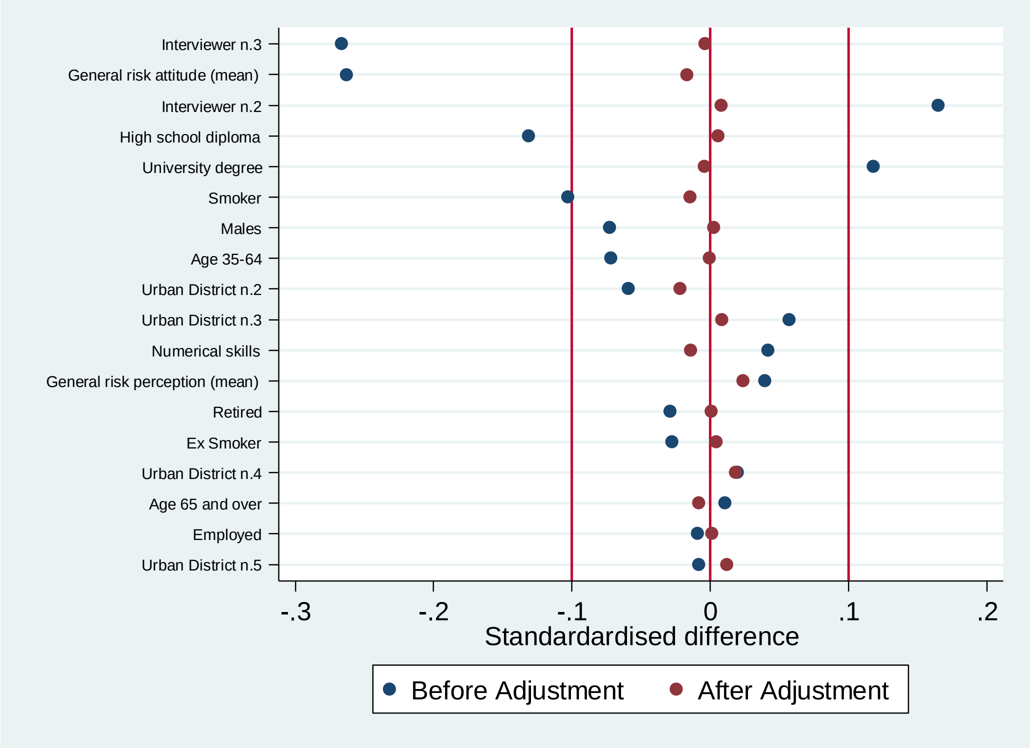


Figure A2. Standardized differences between the two experimental groups for each covariate included in the Propensity Score model, before and after adjustment through IPTW.
