# Supplementary material for: Comparison of two statistical indicators in communicating epidemiological results to the population: a randomized study in a high environmental risk area of Italy
Source: BMC Public Health. 2019 Jun 11;19:733. doi: 10.1186/s12889-019-7003-y (PMC6560769; doi:10.1186/s12889-019-7003-y)
Supplement: Supplementary file 7 — Figure A3. Degree of concern for cancer mortality: estimated differences between TNH and % excess at the quantiles of the outcome distribution (p = 0.1, 0.2, 0.3, 0.4, 0.5, 0.6, 0.7), and corresponding 95% confidence intervals (question R3). (PDF 43 kb) [file 12889_2019_7003_MOESM7_ESM.pdf]

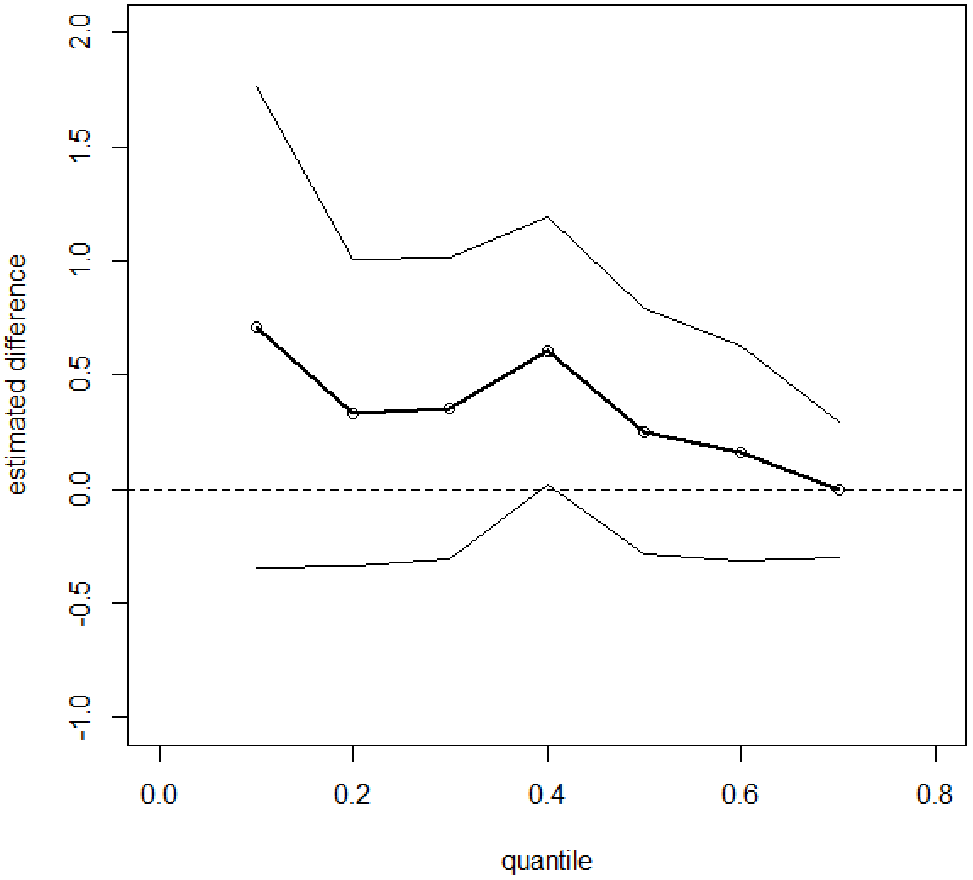

Figure A3. Degree of concern for cancer mortality: estimated differences between TNH and % excess at the quantiles of the outcome distribution (p=0.1, 0.2, 0.3, 0.4, 0.5, 0.6, 0.7), and corresponding 95% confidence intervals (question R3).
