# Supplementary material for: Comparison of two statistical indicators in communicating epidemiological results to the population: a randomized study in a high environmental risk area of Italy
Source: BMC Public Health. 2019 Jun 11;19:733. doi: 10.1186/s12889-019-7003-y (PMC6560769; doi:10.1186/s12889-019-7003-y)
Supplement: Supplementary file 8 — Figure A4. Degree of concern for cancer mortality: estimated differences between the probabilities of a degree of concern larger than the cut-off under TNH and under % excess (cut-off = 2, 3, …, 9), with the corresponding 95% confidence intervals (question R3). (PDF 43 kb) [file 12889_2019_7003_MOESM8_ESM.pdf]

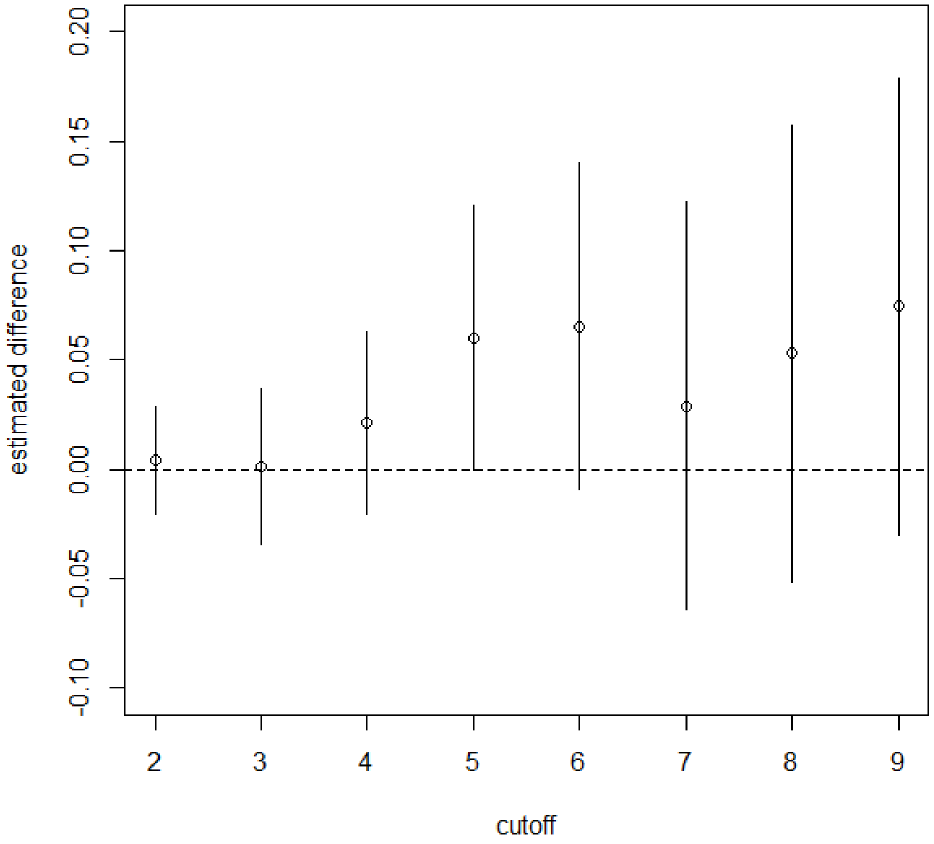

Figure A4. Degree of concern for cancer mortality: estimated differences between the probabilities of a degree of concern larger than the cut-off under TNH and under % excess (cut-off=2, 3,…, 9), with the corresponding 95% confidence intervals (question R3).
