# Supplementary material for: Comparison of two statistical indicators in communicating epidemiological results to the population: a randomized study in a high environmental risk area of Italy
Source: BMC Public Health. 2019 Jun 11;19:733. doi: 10.1186/s12889-019-7003-y (PMC6560769; doi:10.1186/s12889-019-7003-y)
Supplement: Supplementary file 9 — Table A2. Ranking sexual gland cancer, thyroid cancer and lung cancer mortalities according to the degree of concern (from 1: high concern, to 3: low concern). Causal effect of expressing the results in terms of TNH versus % excess (question R4), by education level and mathematical skills. (PDF 18 kb) [file 12889_2019_7003_MOESM9_ESM.pdf]

Table A2. Ranking sexual gland cancer, thyroid cancer and lung cancer mortalities according to the degree of concern (from 1: high concern, to 3: low concern).

Causal effect of expressing the results in terms of TNH *versus* % excess (question R4), by education level and mathematical skills.

		ACE Mean difference	95% CI	P	PO % excess	95% CI
Low mathematical skills[a]	Rank assigned to sexual glands cancer mortality	-0.09	(-0.34 ; 0.15)	0.468	2.30	(2.10 ; 2.50)
	Rank assigned to thyroid cancer mortality	0.20	(-0.16 ; 0.55)	0.276	2.27	(1.96 ; 2.58)
	Rank assigned to lung cancer mortality	-0.11	(-0.39 ; 0.18)	0.472	1.43	(1.18 ; 1.68)
High mathematical skills[a]	Rank assigned to sexual glands cancer mortality	-0.17	(-0.34 ; 0.00)	0.057	2.26	(2.15 ; 2.37)
	Rank assigned to thyroid cancer mortality	0.18	(0.00 ; 0.35)	0.048	2.23	(2.10 ; 2.37)
	Rank assigned to lung cancer mortality	-0.01	(-0.17 ; 0.16)	0.938	1.50	(1.38 ; 1.62)
Intermediate school diploma or lower	Rank assigned to sexual glands cancer mortality	-0.05	(-0.28 ; 0.18)	0.667	2.19	(2.04 ; 2.34)
	Rank assigned to thyroid cancer mortality	0.09	(-0.15 ; 0.33)	0.484	2.41	(2.22 ; 2.59)
	Rank assigned to lung cancer mortality	-0.04	(-0.27 ; 0.20)	0.759	1.40	(1.24 ; 1.56)
High school diploma or higher	Rank assigned to sexual glands cancer mortality	-0.21	(-0.41 ; -0.01)	0.039	2.28	(2.16 ; 2.41)
	Rank assigned to thyroid cancer mortality	0.24	(0.04 ; 0.45)	0.018	2.16	(2.01 ; 2.31)
	Rank assigned to lung cancer mortality	-0.03	(-0.24 ; 0.17)	0.753	1.56	(1.40 ; 1.71)

[a] Low mathematical skills: No right answer at questions evaluating numerical skills; High mathematical skills: At least one right answer at questions evaluating numerical skills.

ACE: Average causal effect.

95% CI: 95% Confidence Interval.

p: p-value

PO % excess: Potential outcome under the % excess questionnaire.
